# Modular intervention to improve paternal involvement and support for better infant and young child feeding in a district of coastal South India:  a randomized controlled trial protocol

**DOI:** 10.12688/f1000research.36376.1

**Published:** 2021-02-17

**Authors:** Prasanna Mithra, Bhaskaran Unnikrishnan, Rekha Thapar, Nithin Kumar, Ramesh Holla, Priya Rathi

**Affiliations:** 1 Department of Community Medicine, Kasturba Medical College, Mangalore, Manipal Academy of Higher Education, Manipal, India

**Keywords:** IYCF, father, involvement, module, child, development

## Abstract

**Background**: The major determinant to the well-being of infants and young children (IYC) is their feeding practices. These practices are the responsibility of both parents, meaning that fathers have an equal role to mothers. Fathers’ involvement can have an impact on the overall health of the children. Despite this, paternal involvement towards IYC feeding (IYCF) have not been studied adequately.

**Methods**: This randomized control trial (n=120) will be conducted among fathers of infants (children aged <1 year) and young children (children aged 12-23 months) in selected households in Dakshina Kannada District of the southern Indian State of Karnataka. The study will be conducted after an initial baseline assessment on awareness, attitude and involvement of fathers in IYCF. Fathers with scores less than the 50 ^th^ percentile in the practice component will be categorized as fathers with poor involvement and will be potential participants for the trial. A visual module will be developed and validated for improving paternal involvement in IYCF. Using a simple randomization technique, the participants will be allocated to modular intervention and control group (1:1 allocation). Each participant in the intervention arm will be visited once a month to implement the module, for six months on a one-to-one basis. Following the intervention, a post-test assessment will be done for both groups to measure the level of paternal involvement in IYCF.

**Ethics and dissemination**: Approval has been obtained from the Institutional Ethics Committee of Kasturba Medical College, Mangalore, India. The dissemination plans include scientific conferences and publication in scientific journals.

**Registration**: The study is registered with Clinical Trial Registry of India ( CTRI/2017/06/008936).

## Introduction

Feeding practices are one of the major determinants of the survival and wellbeing of children ^1^. Providing good nutrition to a child is the responsibility of both parents. Fathers are proficient caregivers; their positive involvement along with mothers in child feeding and rearing is associated with overall health outcomes ^2^. 

The currently available literature points towards the good impact of fathers’ involvement in infants and young children feeding (IYCF) and rearing on the health of their children ^3^. Also, the manner in which fathers assist and support their spouses in terms of child nutrition determines long-term effects ^4^. It has been documented that such positive behaviour from fathers results in a lower BMI among adolescent girls ^4^. Several strategies have been tried to enhance IYCF, including breastfeeding, such as establishing standards in providing maternity services through building public awareness using mass media, peer-support interventions, and health care provider driven initiatives providing maternal support ^5^. Observational studies have documented that the early provision of support from fathers and their active participation in breastfeeding practices, which could happen with adequate knowledge and a positive attitude, had effects on the initiation and overall duration of breastfeeding ^5^.

There are many socio-demographic factors playing an important role in paternal involvement in infant feeding practices, but there is lack of evidence to suggest the extent and determining level of paternal involvement ^6– 8^. Also, the module-based interventions for fathers to enhancing their involvement in IYCF have not been tested.

There are not many documented studies from India in this regard. This study would provide insight about the above stated priority areas and help investigators to develop strategies to enhance infant and young child nutrition, provide a modular intervention to fathers on IYCF, and then evaluate the intervention.

### Review of literature

Fathers play an important supporting role in overall maternal and child health ^9^ and their views of child-feeding practices influence development of the child and eating behaviour ^10– 12^. As a result of changes in societal norms, fathers’ involvement in childcare has increased compared to the level a few years ago. Increased involvement is influenced by several sociocultural, behavioral, hormonal, and neural factors ^13^. With the birth of a first child, a man enters the stage of fatherhood; which is a crucial and challenging period ^14^. If this entry to fatherhood becomes smoother, then the likelihood that the father being more involved in childcare is higher. This can happen with adequate knowledge transfer to the fathers of young children and prospective fathers ^13^. However, there are limited studies that have been done among fathers regarding their involvement in IYCF ^11^. In the Indian scenario, the evidence related to paternal involvement and measures for its enhancement is scant. The focus of interventions and studies related to child feeding are mostly on mothers, since mothers spend the most amount of time with children than anyone else in the family ^10^.

Across different regions of the world, studies done on fathers of young children were mostly of exploratory nature to assess the factors determining IYCF practices, qualitative assessments, couple-oriented interventions, family-based interventions, *etc*. This limited involvement from the fathers’ side were influenced by several region- and culture-specific challenges they faced ^3, 9, 15, 16^. However, in countries like the UK, it was made a strategic priority to enhance breast feeding practices and it was realized that this could be achieved with a “strong social support from their partner” ^17^. It was also felt that fathers need informed guidance in supporting their spouses on breastfeeding and IYCF ^17^. There is no consensus on measuring male involvement and few beliefs exist that imply male involvement could be a threat to women’s empowerment and autonomy in terms of child rearing practices ^18^. Across the studies, there were felt needs from mothers and fathers of the young children regarding increasing the paternal involvement in the child feeding and care. Behaviour Change Communication strategies have been frequently used among mothers. Such strategies, when centered around both the parents have a higher effect on the father’s knowledge and involvement in child feeding, as compared to targeting mothers only. An increase in knowledge, however, may not have the desired impact on IYCF practices ^19^. 

Maycock *et al.* carried out a randomized control trial (RCT) in Perth, Western Australia, known as the Fathers Infant Feeding Initiative (FIFI Study). The trial evaluated the effect of an antenatal paternal education session and spousal support during the postnatal period, targeted at fathers during the antenatal and postnatal period of their spouses. The study reported a significant increase in breastfeeding rate at 6 weeks. They also reported a correlation between a higher age of fathers and a high socio economic status with higher rates of breastfeeding at six weeks ^20^. Abdullahi *et al.*, in Somalia, reported a quasi-experimental study in 2019, commissioned by Save the Children International (SCI), aimed at assessing the effects of peer counselling by Mother-to-Mother (M2M) and Father-to-Father (F2F) support groups on IYCF practices. They observed that there was IYCF knowledge growth, improvement in practices of breastfeeding and diet diversity among intervention arms. Since the follow up duration was short, there was no noticeable change in the nutritional status of children ^21^. 

In Japan, Ito *et al.* reported an inverse relation between infant care by fathers and breastfeeding during the early infancy (first 6 months of life); fathers’ likelihood of involvement was higher with higher education status, being unemployed and nonsmoking ^3^. Pisacane *et al.*, in Naples, Italy, investigated the effect of providing support to fathers in identifying their role in IYCF and teaching them to take care of most common lactation problems. They found that this intervention would result in higher rates of full breastfeeding at 6 months ^5^. Sherriff *et al.*, in Southern England, studied the main attributes of father support in relation to breast feeding. They identified knowledge, attitude and involvement in decision-making as key factors towards successful breastfeeding practice ^22^. 

In rural Kenya, the quasi-experimental study with pre- and post-test observations conducted by Thuita *et al.*, studied the impact of engaging fathers or grandmothers in improving diets of mothers and IYCF feeding practices through “parallel peer education dialogue groups with fathers and grandmothers.” They reported success in bringing increased paternal engagement in IYCF ^23^. 

One study conducted among 210 father-young child dyads in Bangalore, India, in 2020, by Inbaraj *et al.*, aimed to “explore paternal child-feeding patterns, their involvement in feeding, and its association with level of malnutrition in the slums”. The overall involvement in child feeding and rearing was shown by nearly half of the study participants. Religion, family types, and individual income were factors associated with poor involvement of fathers in child feeding ^10^. 

In 2014, Khandpur *et al.* reviewed the existing evidence on child feeding research in 20 studies, which included fathers’ feeding practices and their characteristics and correlations. They included studies which reported the feeding practices of fathers and/or primary care givers towards children aged up to 18 years. Most of the studies reported self-reported child feeding practices without a specific paternal validation. There were also studies reporting fathers pressuring their children to eat. Paternal and maternal feeding practices varied, wherein fathers monitored less and put more limitation to access to child’s food in comparison to mothers. Body fat of children was associated with fathers’ feeding practices ^11^. This review also brought out the paucity of literature on fathers’ child feeding practices.

### Aim of the study

To assess the effectiveness of a module-based intervention for improving the paternal involvement in IYCF.

### Objectives

1. To design and develop an educational module for the fathers on improving father’s IYCF practices and support.

2. To evaluate the effectiveness of module in improving the level of paternal involvement in IYCF

## Methods

Version: 1.0 (22-12-2020).

### Background information of the study area

The study area will be in the Dakshina Kannada District in the southern part of Karnataka State in South India. Regarding health care facilities, it is a progressing area. According to the 2011 Census report, the population of this area was 2,083,625, with a literacy rate of 88.62% ^24^. The current study is being done by the Department of Community Medicine, Kasturba Medical College, Mangalore, Manipal Academy of Higher Education, Manipal, India.

### Study setting

Community healthcare settings in Dakshina Kannada District, Karnataka State, India.

### Study design

The study will be a unblinded parallel group RCT. This study will follow the Consolidated Standards of Reporting Trials (CONSORT) ^25, 26^. The study flow according to CONSORT guidelines is depicted in the *Extended data* ^27^. This study protocol is reported along with Standard Protocol Items: Recommendations for Interventional Trials (SPIRIT) Guidelines ^28^. A completed SPIRIT checklist can be found in the *Reporting guidelines* ^27^.

### Study population

Fathers attending to infants (children aged <1 year) and young children (children aged 12–23 months) in selected households belonging to the geographical areas covered by public health care institutions of Dakshina Kannada District will be included. Households will be identified through records maintained in at public health care institutions. At the time of visit to their houses, their identity will be verified, along with confirmation of the age of their child.

### Sample size

With anticipated improvement in the paternal involvement of IYCF following the modular intervention as 15%, 80% power, 95% Confidence Interval, 1:1 allocation and along with addition of 20% non-response error, the total sample size will be **60** subjects.

### Study duration

The study will be carried out for a total period of one year (between January 2020 and December 2020).

### Eligibility criteria

Fathers of infants and young children with poor involvement in IYCF in households in Dakshina Kannada District will be selected for this study. Initial assessments of all fathers from selected households (see *Study population)* will be conducted to assess fathers’ knowledge, attitude and practices towards IYCF (see questionnaire in *Extended data* ^27^). Fathers with scores in the lower 50 ^th^ percentile in the practice domain will be categorized as fathers with poor involvement in IYCF and will be eligible for inclusion in the RCT.

Fathers who score higher than the 50 ^th^ percentile will be excluded from taking part in the RCT. In addition, fathers who are not available for interventions despite three attempts of contact will be excluded.

To promote retention of the participants, visits will be made to them at a convenient time and location. Intention to treat analysis will be performed (as mentioned in *Data analysis*).

### Sampling strategy and randomization

Based on the findings from initial assessment at the district for level of knowledge, attitude and practices towards paternal involvement in IYCF, the areas with fathers of infants with poor IYCF practices will be mapped for visits at their households. Selected eligible participants will be allocated to modular intervention and control groups. A simple randomization technique with 1:1 allocation will follow. A lottery technique will be used for randomization, until the needed sample size is reached, with the help of Microsoft Excel software. The principal investigator will generate the sequence for randomization and another author (NK) will enroll the participants according to this sequence. The research assistants will implement the modular intervention.

### Intervention procedure

An IYCF improvement module that has been developed as an easy to use flip chart with a blend of pictures and textual materials has been produced (IYCF module, *Extended data*: ^27^), which included the basic aspects of infant and child nutrition and feeding: (i) assisting in feeding; (ii) changing diapers; (iii) giving a bath to the child; (iv) putting the child to sleep; (v) playing with the child and (vi) taking the child outside the home, (e.g. - for a walk).

The module was developed based on the available literature and brainstorming sessions with the researchers. The module will be implemented in the local language (Kannada).

This module will be administered to the intervention group only. The control group will receive regular care at the study centers, which is routinely made available to them whenever they visit health centres or when health workers visit them. This will not affect the ultimate results of the study. The administration of the module to the intervention group will be done monthly for six months. Both groups will be followed up at the end of six months, during which they will be assessed for the level of involvement in IYCF, which is the same method as that of pre-assessment (see questionnaire, *Extended data* ^27^).

### Instruments for data collection

Pre-tested, validated, semi-structured proforma will be used both pre- and post-intervention (questionnaire, *Extended data* ^27^). The instrument will include the demographic details of the participants and Likert scores in these domains: knowledge, attitude and practices (involvement) towards IYCF.

### Data collection methodology

Necessary permissions are taken from the Head of the Institute and District Health Authorities. Two Research Assistants will be recruited from the field of Social Work, with communication skills and ability to interact with the population using the local language. After their training on implementation of the module, they will visit the study participants for the implementation.

The fathers accompanying the infants will be approached and will be provided with the participant information sheet ( *Extended data* ^27^). Written informed consent will be taken from each of them by the research assistants and they will be allocated to one of the intervention groups and one-to-one interaction for module implementation will be followed as stated above. The assessment at six months will be done by the research assistants for both the groups with respect to knowledge, attitude and involvement (practices) in their IYCF.

### Data management

All the data collected in the field will be managed at the central coordinating site, independent of funding organization. A separate Data Monitoring Committee (DMC) will not be constituted. The filled proforma will be edited for inadvertently missing information, related to the demographic component and the participants will be contacted and for missing details. The variables will be coded and entered in IBM SPSS Statistics for Windows (Version 25.0. Armonk, NY: IBM Corp). From this database, 10% of the data will be randomly chosen to validate against the proforma. If the error rate is >0.3%, then the data re-entry will be performed. The data forms will be accessible only to the study staff. The entered data will be secured with a password protected access. The monitoring of data will be undertaken by the administrative staff of the study department and they are not part of the study.

### Data analysis

Results will be expressed as proportions, mean (with standard deviation), median (with interquartile range), using appropriate tables and figures. We will follow intention to treat analysis (ITT). The comparison for the continuous variables will be done using the “t” tests. For comparison across the groups, Chi square tests and Multiple Logistic Regression will be used and p<0.05 will be considered statistically significant.

### Outcomes

The level of involvement of fathers in IYCF, which is of paramount importance to the overall nutrition of children, and extent to which the modules will achieve improvement. In addition, change in knowledge and attitude towards IYCF will be assessed at the end of six months of intervention. The outcomes assessed will be knowledge scores, attitude scores and practice (involvement) scores obtained through the proforma.

### Implications for future research

The current research findings would further be emulated in other regions of the country and elsewhere. Also, the long-term impact of the modular interventions would attract future studies. This in turn would benefit the practitioners and policy makers in enhancing the paternal involvement in IYCF and planning the interventions accordingly. 

### Ethical consideration and dissemination

The study protocol has been approved by the Institutional Ethics Committee (IEC) of Kasturba Medical College, Mangalore, Manipal Academy of Higher Education, Manipal, India. Participants will be given an information letter, and written informed consent will be taken from each of them. All the information collected will be kept confidential. The study has been registered with the Clinical Trial Registry of India ( CTRI/2017/06/008936) ^29^.

The findings of this study will be disseminated through presentations in scientific conferences and scientific journals. Brief policy notes will be prepared and shared with local health authorities.

We will use the CONSORT guidelines, as mentioned in *Study design*, to report the study flow and findings ^26, 30^. For describing the methodology of this trial, we will use the TIDieR Checklist ^31^.

The IEC guidelines of study institute do not permit sharing of research data with any external agency, because they contain personal information and could lead to breach of confidentiality, assured to the participants. However, on personal request and appropriate justification, the data can be obtained from the corresponding author.

## Current status of the study

The study has completed recruiting the participants and four rounds of monthly modular interventions have been administered.

## Data availability

### Underlying data

No data is associated with this article.

### Extended data

Open Science Framework: Modular intervention to improve paternal involvement and support for better infant and young child feeding (IYCF) in a district of coastal South India - A randomized controlled trial (Protocol), https://doi.org/10.17605/OSF.IO/4UB7G ^27^.

This project contains the following extended data:

- Informed consent form and information sheet
- Questionnaire
- IYCF module

### Reporting guidelines

Open Scientific Framework: SPIRIT checklist and CONSORT flowchart for ‘Modular intervention to improve paternal involvement and support for better infant and young child feeding (IYCF) in a district of coastal South India - A randomized controlled trial (Protocol)’, https://doi.org/10.17605/OSF.IO/4UB7G ^27^.

Data are available under the terms of the Creative Commons Attribution 4.0 International license (CC-BY 4.0).
