# Supplementary material for: Modular intervention to improve paternal involvement and support for better infant and young child feeding in a district of coastal South India:  a randomized controlled trial protocol
Source: F1000Res. 2021 May 10;10:121. Originally published 2021 Feb 17. [Version 2] doi: 10.12688/f1000research.36376.2 (PMC8160583; doi:10.12688/f1000research.36376.2)
Supplement: Peer review history

# Peer review: Reviewer response for version 2

**DOI:** 10.5256/f1000research.56456.r85032

**Published:** 2021-05-27

**Authors:** Amutha Ramadas

**Affiliations:** 1 Jeffrey Cheah School of Medicine and Health Sciences, Monash University Malaysia, Bandar Sunway, Malaysia

The paper has been improved upon the previous reviewer's comments. I have a few additional minor comments for the authors to address:

1. Provide a simple clarification within methodology as to why the age groups (<1yr & 12-23 mo) were chosen.
2. Provide a sample size formula or cite the software that was used to calculate the sample size. 
3. Sampling strategy has been adequately explained. 
4. What are the regular care given to the control group? Will the intervention group receives the same care (and then receive the additional intervention)? 

Is the study design appropriate for the research question?

Yes

Is the rationale for, and objectives of, the study clearly described?

Yes

Are sufficient details of the methods provided to allow replication by others?

Yes

Are the datasets clearly presented in a useable and accessible format?

Not applicable

Reviewer Expertise:

Nutrition

I confirm that I have read this submission and believe that I have an appropriate level of expertise to confirm that it is of an acceptable scientific standard.

# Peer review: Reviewer response for version 2

**DOI:** 10.5256/f1000research.56456.r85027

**Published:** 2021-05-11

**Authors:** Haladi Sudhir Prabhu

**Affiliations:** 1 Department of Community Medicine, Father Muller Medical College, Mangalore, India

The changes made are satisfactory.

Is the study design appropriate for the research question?

Yes

Is the rationale for, and objectives of, the study clearly described?

Yes

Are sufficient details of the methods provided to allow replication by others?

Partly

Are the datasets clearly presented in a useable and accessible format?

Not applicable

Reviewer Expertise:

Public health, community Medicine, child health and immunization

I confirm that I have read this submission and believe that I have an appropriate level of expertise to confirm that it is of an acceptable scientific standard.

# Peer review: Reviewer response for version 1

**DOI:** 10.5256/f1000research.39438.r79720

**Published:** 2021-03-04

**Authors:** Haladi Sudhir Prabhu

**Affiliations:** 1 Department of Community Medicine, Father Muller Medical College, Mangalore, India

The study is designed to address an important research question considering the societal context of the current paternal role and means to strengthen it for infant care and feeding through a modular intervention targetting fathers. The authors have done an extensive review on the topic and have designed an extremely sound study protocol to ensure generation of good scientific data for policy and program on child care.

 However, elaborating or modifying a few of the points below may make the protocol more robust prior to begining data collection:

 1. Methods section -

- Study population is being mentioned to be taken from households covered under public health care institutions of Dakshina kannada district. Sampling technique and enrollment is not being mentioned. It is not clear how many such institutions would constitute the primary sampling unit, will there be equal representation of locality (urban,rural) partners of working mothers, socio-demographic and culturally diverse population especially socially deprived communities being enrolled and how would researchers ensure data enrollment complete. Is it conveneient sampling till sample size is achieved?
- Study duration is being mentioned as January 2020 to December 2020 and data collection methodology mentions permission from District health authorities. If data collection has not started it would be better to consider seeking permission from Indian council of medical research as per the circular sent by Directorate of Health and welfare services, Karnataka number NVBDCP/ENT04/2019-20 dated 8/4/2019 mentioning that only ICMR is competent authority to approve community based interventions for private medical college research projects in community area and not district health officer of the district.
- Eligibility criteria: fathers scoring less than 50th percentile will be implied exclusion need not be stated again.
- Intention to treat analysis can be removed from methods section.

2. Intervention procedure -

- Module development technique is not clearly stated - was it through Delphi or any other specific methods and who were the researchers who were involved? Module has images from google images and shutterstock.com which may be under copyright or royalty free clause. Kindly verify due permissions and regulations for the use.
- Even content of the module may need elaboration on specific paternal role for example sleep component mentions only sleep time and images which indicate baby being sleepy, the role of father getting involved is not stated explicitly similarly immunization and growth monitoring doesnt include Thaayi card (mother and child protection card) or immunization card which should be known to father as a go to chart when it comes to vaccination and growth and development details as well as picking up warning signs for poor milestones.

3. CONSORT flow diagram mentioned can be more precise for exclusion, intervention and final outcome process.

 4. Study questionnaire has a mix of Likert, open ended and closed ended questions. If self-administered it may need to be told in detail to study participants before filling up the responses.

 5. Study outcomes and implications - only fathers knowledge, involvement is being assessed at 6 months assessing the health status, growth and development milestones of infant might be better outcome variables to look and understand when it comes to generating evidence.

 6. DK district having higher literacy and less Infant mortality and malnutrition cases among children study may have generalizability issues for the rest of the districts of Karnataka or India.

 7. Informed consent document and patient information sheet should have more details on what is a modular intervention, why and how were the study particpants selected and included in the final trial subject population, their expectations throughout the study period, details on outcome assessment should be clearly stated to study subjects.

Is the study design appropriate for the research question?

Yes

Is the rationale for, and objectives of, the study clearly described?

Yes

Are sufficient details of the methods provided to allow replication by others?

Partly

Are the datasets clearly presented in a useable and accessible format?

Not applicable

Reviewer Expertise:

Public health, Community Medicine, Child health and immunization

I confirm that I have read this submission and believe that I have an appropriate level of expertise to confirm that it is of an acceptable scientific standard, however I have significant reservations, as outlined above.

# Response

**Published:** 2021-05-01

**Authors:** Prasanna Mithra

**Affiliations:** Kasturba Medical College, Mangalore, India

Reviewer Comments; followed by the response (in bold) by Authors:

 The study is designed to address an important research question considering the societal context of the current paternal role and means to strengthen it for infant care and feeding through a modular intervention targetting fathers. The authors have done an extensive review on the topic and have designed an extremely sound study protocol to ensure generation of good scientific data for policy and program on child care.

**Thank you for the in depth review and constructive comments.**

 However, elaborating or modifying a few of the points below may make the protocol more robust prior to begining data collection:

 1. Methods section -

- Study population is being mentioned to be taken from households covered under public health care institutions of Dakshina kannada district. Sampling technique and enrollment is not being mentioned. It is not clear how many such institutions would constitute the primary sampling unit, will there be equal representation of locality (urban,rural) partners of working mothers, socio-demographic and culturally diverse population especially socially deprived communities being enrolled and how would researchers ensure data enrollment complete. Is it conveneient sampling till sample size is achieved?

**A new paragraph is now added in the “sampling strategy” section for enhanced clarity regarding the enrollment procedure.**

- Study duration is being mentioned as January 2020 to December 2020 and data collection methodology mentions permission from District health authorities. If data collection has not started it would be better to consider seeking permission from Indian council of medical research as per the circular sent by Directorate of Health and welfare services, Karnataka number NVBDCP/ENT04/2019-20 dated 8/4/2019 mentioning that only ICMR is competent authority to approve community based interventions for private medical college research projects in community area and not district health officer of the district.

**At present the study stands completed. However, the permissions from the District Health Authorities were sought well before the enforcement of the above referred rule by Directorate of Health and welfare services, Karnataka State, India. Though, the study was carried out by a Private Institute, it had Department of Science and Technology (Govt of India) as the principal supporter through Indian Institute of Public Health Gandhinagar (Government Institute); which was also the technical partner.**

- Eligibility criteria: fathers scoring less than 50th percentile will be implied exclusion need not be stated again.

**Thank you. The duplicate statement is now removed.**

- Intention to treat analysis can be removed from methods section.

**Thank you. The repetition is removed and the mention of intention to treat analysis (ITT) in the methods section is deleted. The statement about ITT is retained only in “Data analysis” section.**

 2. Intervention procedure -

- Module development technique is not clearly stated - was it through Delphi or any other specific methods and who were the researchers who were involved? Module has images from google images and shutterstock.com which may be under copyright or royalty free clause. Kindly verify due permissions and regulations for the use.

**Technique used for the development of the module was “brainstorming technique”; which is mentioned as “The module was developed based on the available literature and brainstorming sessions with the researchers.” in the “Intervention procedure” section. To enhance clarity, a sentence is added about the details of investigators involved in developing the module.**

**Regarding the images, google images used did not have copyright issues; however, we have mentioned the source of images at relevant locations. For “shutterstock.com” images, we had trial account subscription which would give 10 images free for the use.**

- Even content of the module may need elaboration on specific paternal role for example sleep component mentions only sleep time and images which indicate baby being sleepy, the role of father getting involved is not stated explicitly similarly immunization and growth monitoring doesnt include Thaayi card (mother and child protection card) or immunization card which should be known to father as a go to chart when it comes to vaccination and growth and development details as well as picking up warning signs for poor milestones.

**The research assistants were trained to elaborate and explain these specific role of fathers in putting the baby to sleep and in following up on baby’s immunization. Due to several variety of maternal and child protection cards which are commonly used by the families in this area, they were not included in the module as image except for the standard growth chart. However, these cards were scrutinized by the research assistants in the field.**

 3. CONSORT flow diagram mentioned can be more precise for exclusion, intervention and final outcome process.

**Thank you. CONSORT flow diagram is now made more precise with respect to these characteristics.**

 4. Study questionnaire has a mix of Likert, open ended and closed ended questions. If self-administered it may need to be told in detail to study participants before filling up the responses.

**The data collection tool is research assistant administered. Hence the study participant would not go through the challenge of filing variety of questions.**

 5. Study outcomes and implications - only fathers knowledge, involvement is being assessed at 6 months assessing the health status, growth and development milestones of infant might be better outcome variables to look and understand when it comes to generating evidence.

**Thank you for the suggestions. However, the objective of the intervention is only to target the paternal knowledge, attitude and involvement (practice) towards IYCF. Assessing milestones and growth pattern would have demanded more work force; which was beyond the feasibility range of this interventional study.**

 6. DK district having higher literacy and less Infant mortality and malnutrition cases among children study may have generalizability issues for the rest of the districts of Karnataka or India.

**Thank you. This model of paternal intervention included participants from different strata of society and standard recruitment techniques were being followed to minimize the challenges of generalization. Success of this model could also be tested in other parts of the country or elsewhere. Moreover, this trial also provides a guide to feasibility aspects.**

 7. Informed consent document and patient information sheet should have more details on what is a modular intervention, why and how were the study particpants selected and included in the final trial subject population, their expectations throughout the study period, details on outcome assessment should be clearly stated to study subjects.

**The entire consenting process involved a detailed conversation between the research assistant in the field and the study participant. Therefore, the participant information sheet provided herewith is one part of the consenting process and the conversation uniformly involved explanations about the nature of intervention, frequency of contact, post intervention assessment and parameters which would assessed and measured.**
